# A functional variant on 20q13.33 related to glioma risk alters enhancer activity and modulates expression of multiple genes

**DOI:** 10.1002/humu.24134

**Published:** 2020-11-22

**Authors:** Mourad Wagdy Ali, C. Pawan K. Patro, Jacqueline Jufen Zhu, Christopher H. Dampier, Sarah J. Plummer, Cem Kuscu, Mazhar Adli, Ching Lau, Rose K. Lai, Graham Casey

**Affiliations:** ^1^ Department of Public Health Sciences, Center for Public Health Genomics University of Virginia Charlottesville Virginia USA; ^2^ Department of Neurology, Keck School of Medicine University of Southern California Los Angeles California USA; ^3^ The Jackson Laboratory for Genomic Medicine Farmington Connecticut USA; ^4^ Department of Surgery, James D. Eason Transplant Research Institute University of Tennessee Memphis Tennessee USA; ^5^ Department of Obstetrics and Gynecology, Robert Lurie Comprehensive Cancer Center Feinberg School of Medicine at Northwestern University Chicago Illinois USA; ^6^ Departments of Neurology and Preventive Medicine, Keck School of Medicine University of Southern California Los Angeles California USA

**Keywords:** 20q13.33, enhancer, functional variant, GBM, glioma, GWAS

## Abstract

Genome‐wide association studies (GWAS) have identified single‐nucleotide polymorphisms (SNPs) associated with glioma risk on 20q13.33, but the biological mechanisms underlying this association are unknown. We tested the hypothesis that a functional SNP on 20q13.33 impacted the activity of an enhancer, leading to an altered expression of nearby genes. To identify candidate functional SNPs, we identified all SNPs in linkage disequilibrium with the risk‐associated SNP rs2297440 that mapped to putative enhancers. Putative enhancers containing candidate functional SNPs were tested for allele‐specific effects in luciferase enhancer activity assays against glioblastoma multiforme (GBM) cell lines. An enhancer containing SNP rs3761124 exhibited allele‐specific effects on activity. Deletion of this enhancer by CRISPR‐Cas9 editing in GBM cell lines correlated with an altered expression of multiple genes, including *STMN3, RTEL1*, *RTEL1‐TNFRSF6B*, *GMEB2*, and *SRMS*. Expression quantitative trait loci (eQTL) analyses using nondiseased brain samples, isocitrate dehydrogenase 1 (*IDH1*) wild‐type glioma, and neurodevelopmental tissues showed *STMN3* to be a consistent significant eQTL with rs3761124. *RTEL1* and *GMEB2* were also significant eQTLs in the context of early CNS development and/or in *IDH1* wild‐type glioma. We provide evidence that rs3761124 is a functional variant on 20q13.33 related to glioma/GBM risk that modulates the expression of *STMN3* and potentially other genes across diverse cellular contexts.

## 1 INTRODUCTION

Gliomas are the most common form of brain cancer, accounting for around 13,000 deaths in the USA each year (Bondy et al., 2008; Ostrom et al., 2015). Gliomas are a heterogeneous group of tumors, which are typically associated with a poor prognosis. The most common type of glioma, glioblastoma multiforme (GBM), has a median overall survival of only 10–15 months (Bondy et al., 2008).

Genome‐wide association studies (GWAS) of glioma have led to the discovery of at least 25 inherited risk variants (Kinnersley et al., 2018). These 25 loci, in total, are estimated to account for approximately 30% of heritable risk (Melin et al., 2017). The discovery of the biological mechanism underlying these risk variants has the potential to reveal novel insights into glioma development. However, characterization of the biological basis of risk has proven to be challenging, because few index GWAS single‐nucleotide polymorphisms (SNPs) are themselves functional. The emerging picture is that most functional/causal SNPs associated with risk map to enhancers or promoters and lead to an altered gene expression (Biancolella et al., 2014; Fortiniini et al., 2014).

Several GWAS and GWAS meta‐analyses have identified 20q13.33 as a risk locus, especially its association with isocitrate dehydrogenase 1 (*IDH1*) wild‐type or *TERT* (telomerase reverse transcriptase)‐only gliomas. (Eckel‐Passow et al., 2015; Enciso‐Mora et al., 2013; Kinnersley et al., 2015; Labreche et al., 2018; Rajaraman et al., 2012; Sanson et al., 2011; Shete et al., 2009; Walsh et al., 2014; Wrensch et al., 2009; Wu et al., 2019). In the first two GWAS conducted on glioma, several risk variants were identified that reached genome‐wide significance in 20q13.33, with rs6010620 (NC_000020.10:g.62309839A>G) being the common risk SNP between the two studies (Shete et al., 2009; Wrensch et al., 2009). Subsequent GWAS further confirmed 20q13.33 as a risk locus (Kinnersley et al., 2015; Rajaraman et al., 2012), with the most recent and largest Glioma International Case‐Control (GICC) GWAS meta‐analysis (cases: 12,496, controls: 18,190) again confirming the association between a polymorphism in 20q13.33 and glioma, and the top GWAS SNP as rs2297440 (GICC GWAS meta‐analysis *p* = 1.16E−38; NC_000020.10:g.62312299T>C; Melin et al., 2017), where the association was strongest with GBM. These studies reveal that 20q13.33 is among the most consistently validated GWAS locus for glioma/GBM. However, functional variants within the region have yet to be identified.

The top GWAS SNP at 20q13.33, rs2297440, maps to Intron 14 of the regulator of telomere elongation helicase 1 (*RTEL1*). It is unlikely that this SNP is functional/causal, as it does do not map to any functional elements, including enhancers, in astrocytes or cortical tissues (data not shown). We, therefore, tested the hypothesis that the functional/causal SNP(s) in this region were in linkage disequilibrium (LD) with this top GWAS SNP. To identify candidate functional variants within the 20q13.33 GWAS locus, we systemically screened SNPs in LD with rs2297440 that intersected with regulatory elements/enhancers as cataloged in publicly available annotations. Each candidate enhancer/SNP region identified was tested in luciferase reporter assays for SNP‐dependent allele‐specific effects on enhancer activity. Using this approach, we identified a functional SNP rs3761124 that mapped to an enhancer region on 20q13.33. This SNP had allele‐specific effects on enhancer activity in cell‐based luciferase reporter assays. Several candidate target genes of this enhancer were identified after CRISPR‐Cas9 deletion including stathmin 3 (*STMN3*). We further demonstrated that this variant correlated with the expression of several *cis* genes using eQTL analysis, including *STMN3* as the most consistent target gene across different cellular contexts.

## 2 MATERIALS AND METHODS

### 2.1 Region analysis

**Figure 1:**
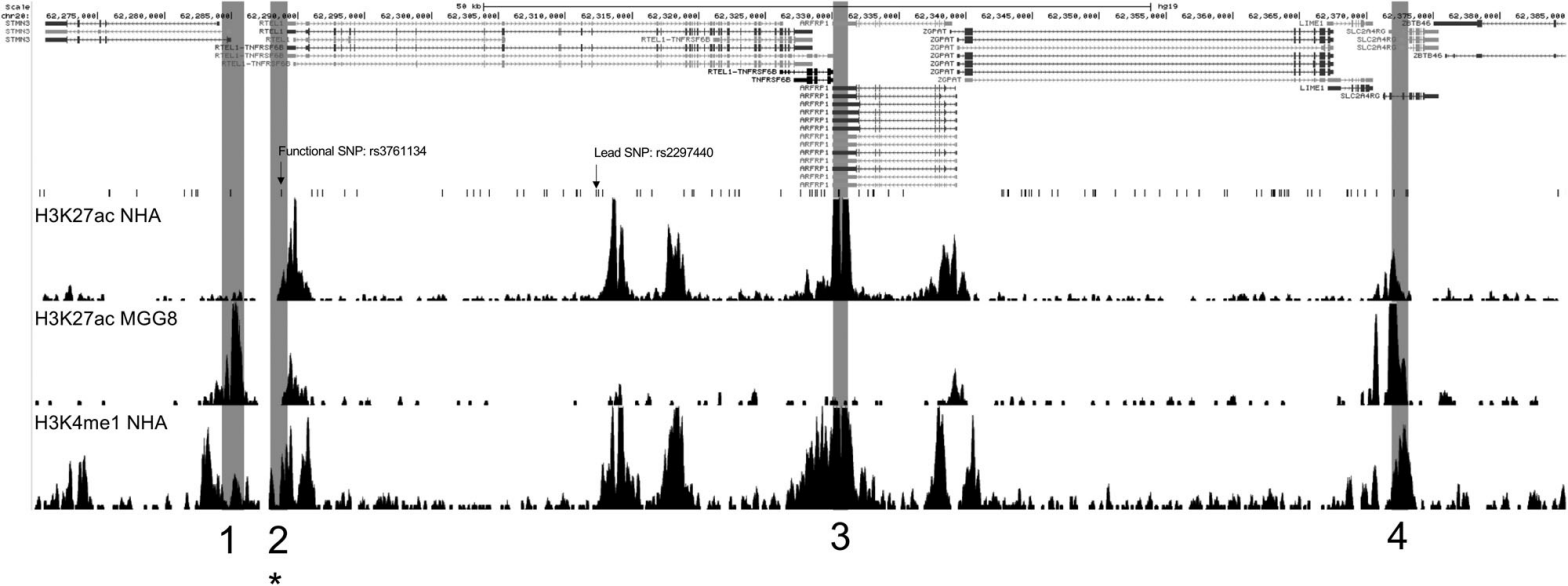
The region of chromosome 20 associated with glioblastoma risk. A detailed view of the region defined by LD block with lead SNP rs2297440, *r* ^2^ > .6 in CEU population, using the UCSC Genome Browser showing putative enhancer elements containing SNPs in LD with rs2297440. SNPs in LD are observed below genes in the region. Histone ChIP‐Seq tracks for H3K27ac from normal human astrocytes (NHA), MGG8 glioblastoma stem cells, and H3K4me1 from NHA aligned below SNPs indicate potential enhancer elements. Region 1 denotes a region with no enhancer activity in luciferase assays. Region 2 denotes the allele‐specific enhancer region, which includes rs3761124 (marked with an asterisk). Regions 3 and 4 denote regions that exhibited enhancer activity but were unaffected by haplotype. It should be noted that the size of the regions tested for enhancer activity is not to scale. LD, linkage disequilibrium; SNP, single‐nucleotide polymorphism; UCSC, University of California Santa Cruz

Several GWAS have shown an association between SNPs mapping to chromosome 20q13.33 and glioma risk (Kinnersley et al., 2015; Melin et al., 2017; Rajaraman et al., 2012; Shete et al., 2009). We identified an LD block of approximately 116 kb on chromosome 20q13.33, which included 120 SNPs (Figure 1) in LD with the lead SNP in the most recent GWAS meta‐analysis (rs2297440; Melin et al., 2017). LD was determined by *r* ^2^ > = .6 in the CEU population. To identify candidate functional SNPs, we used University of California Santa Cruz Genome Browser (http://genome.ucsc.edu/; Kent et al., 2002) to overlay the SNPs in LD with rs2297440 with physiologically relevant histone ChIP‐Seq peaks for H3K4me1 (from Encyclopedia of DNA Elements [ENCODE] normal human astrocyte cell line GSM733710) and H3K27ac (from ENCODE normal human astrocyte cell line GSM733763 and ENCODE GBM cell line GSM1121878; Figure 1). All cloned candidate enhancer regions contained at least one SNP in LD (*r* ^2^ > = .6) with the lead SNP (rs2297440) and coincided with peaks of chromatin marks of enhancers in at least two relevant ChIP‐seq datasets. Additional histone ChIP‐Seq peaks for enhancer elements derived from normal human astrocytes, human glioblastoma cancer stem cells, and human glioblastoma cell lines were also used in these analyses (GSM1121881, GSM894065, GSM1515744, GSM2500170, GSM1121859, and GSM1121869; data are not shown). This analysis resulted in the identification of four candidate enhancer elements that each contained at least one SNP in LD with rs2297440 (Figure 1).

### 2.2 Cell culture

LN‐229 and U‐87 MG GBM cell lines were obtained from American Type Culture Collection. LN‐229 cells were grown in Dulbecco's modified Eagle's medium (DMEM; Thermo Fisher Scientific) supplemented with 5% fetal bovine serum (FBS; Thermo Fisher Scientific) and 1% penicillin/streptomycin, and incubated at 37°C and 5% CO_2_. U‐87 MG cells were grown in DMEM (Thermo Fisher Scientific) supplemented with 10% FBS (Thermo Fisher Scientific) and 1% penicillin/streptomycin, and incubated at 37°C and 5% CO_2_.

### 2.3 Plasmids and luciferase assays

DNA fragments containing alternate alleles of each of the four candidate SNPs/haplotypes were polymerase chain reaction (PCR)‐amplified from normal human genomic DNA and subcloned into Sac I and Xho I restriction enzyme sites (in both orientations) upstream of a thymidine kinase (TK) minimal promoter‐driven firefly luciferase vector (courtesy of Dr. G. A. Coetzee, Van Andel Research Institute) using CloneAmp HiFi PCR Premix and the In‐Fusion HD cloning kit (Takara). Plasmid clones were sequenced by Sanger sequencing (Genewiz) to confirm the presence of the candidate variants and the absence of any PCR amplification‐induced mutations.

A region of 1p31.3, previously shown to have no activity in any of the cell lines, served as the negative control, and a region of 11q23.3, previously shown to have enhancer activity in all of the cell lines, served as the positive control. For enhancer assays, LN‐229 (25 × 10^3^ cells/well) and U‐87 MG cells (7 × 10^4^ cells/well) were seeded into 96‐well plates. Cells were cotransfected with reporter plasmids and constitutively active pNL1.1.TK [Nluc/TK] Vector (Promega) using Lipofectamine 2000 Reagent (Thermo Fisher Scientific) according to the manufacturer's instructions. After 48 h, cells were assayed for luciferase activity using the Dual‐Luciferase Reporter Assay System (Promega), according to the manufacturer's instructions, and measured using a Synergy H1 Microplate Reader (BioTek). To quantify enhancer activity in cells transfected with candidate enhancer elements, luminescence resulting from the transcription and translation of firefly luciferase in the presence of luciferin was measured, and background luminescence in the absence of reagents was subtracted. To control for cell concentration, normalization with luminescence from constitutively active NanoLuc luciferase was performed (i.e., candidate enhancer/constitutive control). To control for assay artifact, normalization with luminescence from cells transfected with clones empirically found to have no enhancer activity was performed (i.e., candidate enhancer/negative control). Measurements for each enhancer were obtained in three wells (i.e., technical replicates) for four clones (i.e., biological replicates) for each of the two alleles observed in human populations (i.e., experimental conditions) on three separate days (i.e., experimental validations) and in two independent cell lines (i.e., experimental validations). For statistical testing, measurements from the two cell lines were considered separately, and regression analysis was performed with generalized estimating equations to account for repeated measurements of clones (Goldhoff et al., 2008; Zeger & Liang, 1986).

### 2.4 CRISPR‐Cas9 genome editing

Upstream and downstream CRISPR gRNAs (guide RNAs) were designed flanking the candidate enhancer regions, using https://www.crisprscan.org/ (Moreno‐Mateos et al., 2015; guide sequences; gRNA1: 5' GCCATGTACGACCTGGGAAC, gRNA2: 5' GCTGCGTGACCGCGCACGGC), and cloned into modified transient expression plasmid from Addgene (42230). Briefly, the plasmid was digested with PstI and self‐ligated to obtain only gRNA expressing plasmid. CACC and AAAC overhangs were added on the 5’ of the oligos (Integrated DNA Technologies) and then self‐annealed to produce inserts. Later, modified plasmid was digested with BbsI to produce a vector that is ligated with annealed oligos in the presence of DNA Ligase IV (New England Biolabs) to produce gRNA expressing plasmids (gRNA1 Forward: 5′CACCGCCATGTACGACCTGGGAAC, gRNA1 Reverse: 5′AAACGTTCCCAGGTCGTACATGGC; gRNA2 Forward: 5′CACCGCTGCGTGACCGCGCACGGC, gRNA2 Reverse: 5′AAACGCCGTGCGCGGTCACGCAGC). The Cas9‐expressing plasmid was purchased from Addgene (41815). LN‐229 and U‐87 MG cells were transfected by gRNA‐ and Cas9‐expressing plasmids (1:3 ratio) using Lipofectamine 2000 (Thermo Fisher). Transfected cells were selected by at least 5‐day treatment with geneticin (1 mg/ml for LN‐229 cells and 0.5 mg/ml for U‐87 MG cells) before DNA and RNA harvesting. Genomic DNA was purified using the QIAamp DNA Mini Kit (Qiagen) and enhancer deletion was confirmed with PCR amplifications (forward primer: 5′ GCCTGACCAACATGATGAAA, reverse primer: 5′ TGGCCAGTGAACCTCACTTC).

### 2.5 Quantitative Real‐Time PCR

RNA was isolated using Trizol reagent (Thermo Fisher) and cDNA was synthesized from 2 μg of total RNA using the High‐Capacity Reverse Transcriptase cDNA Kit (Thermo Fisher Scientific). The quantitative real‐time polymerase chain reaction (RT‐PCR) was performed using Superscript III Kit for RT‐PCR (Thermo Fisher Scientific) and amplified with TaqMan assays for genes mapping within 250‐kb upstream and downstream of SNP rs3761124: *RTEL1* (assay ID: Hs01566915_m1), *RTEL1‐TNFRSF6B* (Hs01548060_m1), Src‐related kinase lacking C‐terminal regulatory tyrosine and N‐terminal myristylation site (*SRMS*; Hs00998384_m1), glucocorticoid modulatory element binding protein 2 (*GMEB2*; Hs00202606_m1), *STMN3* (Hs00274822_m1), ADP ribosylation factor‐related protein 1 (*ARFRP1*; Hs00182389_m1), protein tyrosine kinase 6 (*PTK6*; Hs00966641_m1), SLC2A4 regulator (*SLC2A4RG*; Hs00219920_m1), eukaryotic translation elongation factor 1 alpha 2 (*EEF1A2*; Hs00951278_m1), pancreatic progenitor cell differentiation and proliferation factor (*PPDPF*; Hs01100976_g1), helicase with zinc finger 2 (*HELZ2*; Hs00375688_m1), fibronectin type III domain‐containing 11 (*FNDC11*; Hs01868475_s1), zinc finger CCCH‐type and G‐patch domain containing (*ZGPAT*; Hs00738790_m1), Lck‐interacting transmembrane adapter 1 (*LIME1*; Hs00942226_g1), zinc finger and BTB domain‐containing 46 (*ZBTB46*; Hs01008166_m1), abhydrolase domain‐containing 16B (*ABHD16B*; Hs00607796_s1), DnaJ heat shock protein family (Hsp40) member C5 (*DNAJC5*; Hs01122831_m1), tumor protein D52 like 2 (*TPD52L2*; Hs00900580_g1), and *TBP* (internal control; Hs00427620_m1) in three independent experiments and in triplicate for each RNA preparation on QuantStudio 5 (Thermo Fisher Scientific), and analyzed using GraphPad Prism (version 8.3.0, GraphPad Software; www.graphpad.com). Reactions were normalized using the control gene *TBP*, and calculations were performed according to the $2^{{‐∆∆C}_{\text{t}}}$ method. Fold change in the expression was determined from three independent experimental repeats, each performed in duplicate, unless otherwise noted. Data were analyzed for statistical differences using an analysis of variance, with Bonferroni correction for multiple hypothesis testing. *p < .05; **p < .01; and ***p < .001 indicate the levels of significance.

### 2.6 eQTL mapping

The association between rs3761124 and expression of *cis* genes was evaluated in adult brains without neurological diseases, during early neurological development, and in *IDH1* wild‐type adult glioma. To perform eQTL analyses across these different biological contexts, data sets generated from the CommonMind Consortium (CMC), the University of California, Los Angeles (UCLA) postconception fetal tissues collection, and The Cancer Genome Atlas (TCGA) GBM and lower grade glioma (LGG) cohorts were used.

Approval was obtained from the National Institute of Mental Health to use the control brain dataset of CMC release 1. These data were generated in postmortem brain tissues, previously verified to be free of any neurological diseases. The eQTL analysis included the genotyping and RNA‐seq data of a total of 216 unique individuals of European ancestry. RNA‐Seq FASTQ files of CMC were downloaded from https://www.synapse.org/ (syn2759792) and mapped to hg19 using STAR (Dobin et al., 2013), with Gencode v19 as the reference annotation. FeatureCounts from the Subread package (http://subread.sourceforge.net/) was used to generate gene‐level counts from the aligned reads. All genes included in the analysis had more than five reads across all samples, and less than 10 samples per gene had zero reads. The filtered counts of the target genes were normalized using the variance‐stabilizing transformation in DESeq2 (Love et al., 2014). Genotyping data of rs3761124 were extracted from Illumina Infinium HumanOmniExpressExome array data (plink file), and the alleles were matched to the forward strand of GRCh37 reference genome using the Bcftools fixref plugin (http://www.htslib.org/doc/#publications). Linear regression was used to evaluate the association between rs3761124 and specific target genes discovered by quantitative RT‐PCR, with age, gender, RIN scores, the first three principal components of genotype, and RNA‐seq expression residuals as covariates.

To evaluate the effect of rs3761124 on gene expression during early neurological development, a dataset generated from resources of the UCLA Gene and Cell Therapy core (Walker et al., 2019) was obtained from dbGAP, which approved the use of genotyping and RNA‐seq data of 219 donors of European ancestry (postconception weeks 14−21). RNA‐seq SRA files were converted to FASTQ format using the fastq‐dump utility of the SRAtoolkit v2.10.5 (http://ncbi.github.io/sra-tools/), followed by processing of FASTQ files using the same pipeline (as CMC dataset) to obtain, filter, and normalize gene counts. Data for the functional SNP rs3761124 were extracted from the processed genotype data in PLINK format (Purcell et al., 2007), and the alleles were matched with the forward strand of GRCh37 reference genome using the Bcftools fixref plugin. eQTL mapping was performed in the same way as normal brain tissues using linear regression, except the covariate age that was substituted as gestational age.

To evaluate eQTL after glioma is established, the combined cohort of GBM and LGG from TCGA was used (Ceccarelli et al., 2016). As the 20q13.33 locus is most relevant to the subset of *IDH1* wild‐type glioma, *IDH1* wild‐type subjects were identified in the cBioPortal and corresponding clinical, RNA‐seq BAM files, and preprocessed copy number alteration data were downloaded through the CDG portal. Germline genotype files (Affymetrix SNP 6.0 level 2 data) were downloaded from the GDC Legacy Archive and included a total of 211 *IDH1* wild‐type subjects of European ancestry. Access to controlled TCGA data was approved by dbGAP. Genotyping data were first converted to calls with confidence scores of more than 0.9. Afterward, SNPs were checked for concordance with 1000 Genomes, flipped to the positive strand accordingly, and then SHAPE‐IT and IMPUTE2 were used to phase and impute SNPs on 20q13.33, including rs3761124 (IMPUTE2 info score 0.9; Delaneau et al., 2008; Howie et al., 2009). RNA‐seq BAM files were converted to FASTQ format using SAMtools and BEDtools (Li et al., 2009; Quinlan & Hall, 2010). Downstream processing of FASTQ files was performed as previously mentioned. As the effect of the functional SNP on gene expression can be confounded by segmental and focal copy number variations (CNVs) in tumors, segmental CNV data (Level 3) were also collected from TCGA. Segment means were generated by reverse log2 transformation of the segmented copy number values. Focal‐level CNV values of candidate target genes *STMN3, SRMS, RTEL1, RTEL‐TNFRSF6B*, and *GMEB2* were retrieved from the masked copy number segment files, which were generated using GISTIC2 (Beroukhim et al., 2010). eQTL mapping was performed similar to the CMC and UCLA datasets, with the addition of focal and segmental CNV values as covariates in linear regression analyses.

As all eQTL analyses were hypothesis‐driven and were guided by significant results of quantitative RT‐PCR of *cis* genes following CRISPR‐Cas9 experiments, there was no type‐1 error adjustment of the results.

### 2.7 Colocalization of eQTL and GWAS signals

To provide additional supporting data that the functional SNP rs3761124 is likely responsible for the signals in both GWAS and eQTL analyses, the most concordant eQTL result was colocalized with the GICC GWAS meta‐analysis summary result (case: 12,496, control: 18,190; Melin et al., 2017). The GICC GWAS meta‐analysis result for rs3761124 was highly significant (β = .29 (.02), *p* = 1.3E^‐37^). We used the COLOC software to evaluate the posterior probability that the genetic association with gene expression is driven by the same variant driving the GWAS risk association (termed PP4; Giambartolomei et al., 2014). The method also evaluates if the expression association and disease association are driven by two distinct causal variants (PP3). A high PP4 (>0.8) and low PP3 (<0.2) indicate that a single variant (rs3761124) is responsible for both the GWAS and eQTL signals.

### 2.8 Visualization of Hi‐C chromosome conformation interactions

The 3D Genome Browser (http://3dgenome.org) was used to visualize Hi‐C data in 20q13.33 in GBM cell line G583 (Johnston et al., 2019; Wang et al., 2018). The bait region contained rs3761124, and the browser extracted Hi‐C data centered on the bait region and presented interaction events as peak signals in nearby or distal genomic regions; hence, virtual 4C data were constructed from Hi‐C data, with resolution (bin size) at 5,000 bp.

## 3 RESULTS

### 3.1 Candidate enhancer region characterization

**Figure 2:**
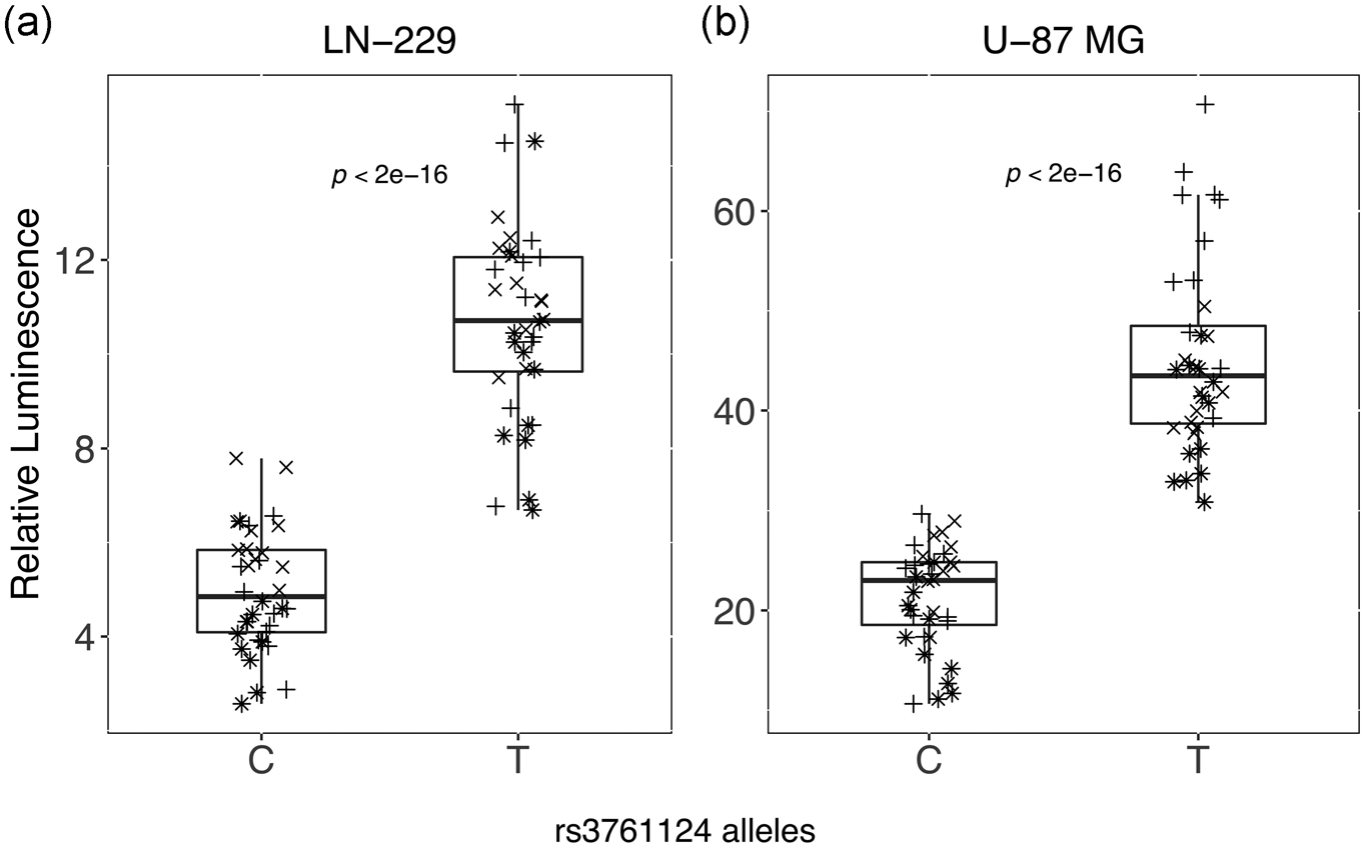
Allele‐specific enhancer activity of enhancer region 2. All enhancer regions seen in Figure 1 were cloned into a luciferase enhancer assay construct and tested for enhancer activity. Here, we show data for enhancer region 2 that includes SNP rs13761124 alleles T and C (four clones for each allele, three independent experiments for each cell line: (+) represents Experiment 1, (×) represents Experiment 2, and (*) represents Experiment 3. The construct with the T allele demonstrated statistically significantly higher activity than the C allele, as shown in box plots from LN‐229 (a) and U‐87 MG cells (b).*p* values represent the probability that the coefficient estimated for allele in the generalized estimating equation model used to test the effect of allele and experiment on relative luminescence would be observed if the true effect was zero. SNP, single‐nucleotide polymorphism

All four putative enhancers shown in Figure 1 were cloned separately into luciferase enhancer activity vectors (Biancolella et al., 2014; Fortiniini et al., 2014), and enhancer activities of different alleles were independently tested with measurements of luminescence after transfection of constructs into two GBM cell lines, LN‐229 and U‐87 MG. Three of the four regions (Regions 2, 3, and 4 in Figure 1) demonstrated enhancer activity in cell lines in at least one orientation (data are not shown). There were a total of six SNPs (*r* ^2^ of ≥ .6 with rs2297440) within these three enhancer regions (rs3761124 [NC_000020.10:g.62288752T>C] in Region 2, rs1291209 [NC_000020.10:g.62330439T>C] and rs1295810 [NC_000020.10:g.62330484G>A] in Region 3, and rs1741708 [NC_000020.10:g.62372041G>T], rs2253823 [NC_000020.10:g.62372956C>T], and rs2253829 [NC_000020.10:g.62373079G>C] in Region 4), but only one, rs3761124, in the enhancer Region 2 (Figure 1) demonstrated allele‐specific effects. rs3761124 is 23.7 kb away from the top GWAS SNP, rs2297440, and is in high LD with it (*r* ^2^ of .92 in the European population). Whereas the candidate enhancer in Region 2 demonstrated activity in luciferase assays in both directions, rs3761124 showed allele‐specific effects on enhancer activity in the forward orientation only (Figure 2), and not the reverse orientation in either cell line (data not shown). The fragment containing the T allele (the reference allele) correlated with higher activity than the fragment containing the C allele (the minor allele) in both of the cell lines and all of the replicates tested. Region 1 did not show enhancer activity in either of the two cell lines tested (Figure 1). rs201497780 (NC_000020.10:g.62284926_62284927delGA) within Region 1 did not demonstrate enhancer activity in cell lines (Figure 1).

### 3.2 CRISPR−Cas9 enhancer disruption

**Figure 3:**
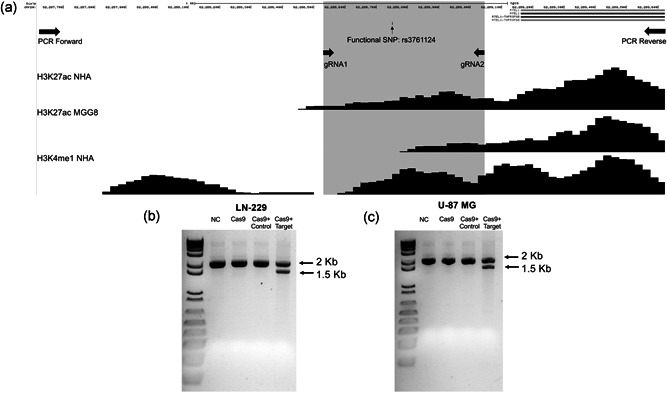
CRISPR−Cas9 genome editing of enhancer region 2 on 20q13.33. (a) Chromosome view of the section of putative enhancer region 2 targeted by CRISPR−Cas9 genome editing technique in the UCSC Genome Browser. The total region (∼2 kb) represents a part of putative enhancer region 2, containing the candidate functional SNP rs3761124, amplified by PCR (using Forward and Reverse primers). The region highlighted in gray represents region (∼0.5 kb) targeted by CRISPR gRNAs (gRNA1 and gRNA2). The 1.5‐kb band in DNA gel electrophoresis demonstrates the targeted deletion of putative enhancer region 2, containing SNP rs3761124, in LN‐229 (b) and U‐87 MG cells (c). Cas9, cells transfected with Cas9 only (no guide RNAs); Cas9+ control, cells transfected with Cas9 vector and gRNA empty vector; Cas9+ target, cells transfected with Cas9 vector and guide RNA target vectors; NC, mock‐transfected parental cells; PCR, polymerase chain reaction; SNP, single‐nucleotide polymorphism; UCSC, University of California Santa Cruz

To provide evidence that the candidate functional SNP rs3761124 identified in our cell‐based luciferase assays correlated with the altered expression of genes mapping in *cis*, we used CRISPR−Cas9 genome editing to delete the region containing SNP rs3761124. The GBM cell lines LN‐229 and U‐87 MG were chosen for these experiments because they both expressed detectable levels of many of the potential target genes in the region and due to the demonstrated enhancer activity of the fragment containing rs3761124 in these cell lines. We designed guide RNAs (gRNA1 and gRNA2) to delete an approximately 500‐bp fragment containing the enhancer and SNP rs3761124 (Figure 3a). Each target sequence was cloned into a gRNA expression plasmid and used together with Cas9 expression plasmids to induce targeted deletion. LN‐229 and U‐87 MG cells were transfected with either Cas9‐only expressing vector, Cas9 vector and gRNA empty vector, or Cas9 vector and guide RNA target vectors. Transfected cells were placed under geneticin selection for at least 5 days, after which DNA was harvested and used to assess the deletion efficiency. Deletion efficiency was measured by PCR using primers designed to amplify an approximately 2‐kb region across the putative enhancer containing the candidate functional SNP rs3761124 (PCR Forward and PCR Reverse in Figure 3a). The cell population with the unedited genome revealed a 2‐kb band, whereas the population with the edited cells revealed two bands: the unedited 2‐kb fragment and the edited 1.5‐kb fragment. Our results show that a large proportion of cells (though not all) in both cell lines were successfully edited (Figure 3b,c). RNA was isolated from the same cellular pools, and the expression of genes within 250 kb in each direction for which a TaqMan assay was available was tested by qPCR.

### 3.3 Gene expression analysis

**Figure 4:**
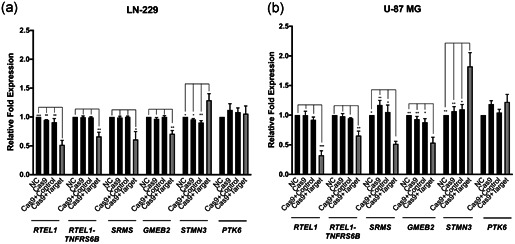
The gene expression changes after CRISPR−Cas9 deletion of Enhancer 2. The genomic region corresponding to enhancer region 2 was targeted for deletion in LN‐229 (a) and U‐87 MG (b) cells using CRISPR−Cas9 technology. Pools of transfected cells were analyzed using qPCR and Taqman gene expression assays for *RTEL1*, *RTEL1‐TNFRSF6B*, *SRMS*, *GMEB2*, *STMN3*, *PTK6*, and *TBP*(control) custom assays in triplicate, in three independent experiments. cas9, cells transfected with Cas9 only (no guide RNAs); Cas9+Control, cells transfected with Cas9 vector and gRNA empty vector; Cas9+Target, cells transfected with Cas9 vector and guide RNA target vectors; NC, mock‐transfected parental cells. Targeting of the region resulted in a decrease in *RTEL1*, *RTEL1‐TNFRSF6B*, *SRMS*, and *GMEB2* expression levels, whereas *STMN3* expression levels increased significantly. *PTK6* expression levels did not change significantly. *p < .05; **p < .01; and ***p < .001 indicate the levels of significance

A number of genes mapping to chromosome 20q13.33 have been implicated in glioma risk (Atkins et al., 2019). We hypothesized that CRISPR−Cas9 mediated disruption of the region containing the candidate functional SNP rs3761124 may affect the expression of multiple genes. To test this hypothesis, we quantified gene expression within 250 kb on either side of the rs3761124 using Taqman qPCR expression assays in CRISPR genome‐edited GBM cell lines and compared expression to mock CRISPR‐edited cells. We observed a statistically significant reduction in the expression of several genes, including *RTEL1*, *RTEL1‐TNFRSF6B* (RTEL1‐TNFRSF6B readthrough [nonsense‐mediated mRNA decay [NMD] candidate]), *SRMS*, and *GMEB2* (Figure 4). We also observed a statistically significant increase in *STMN3* expression. We did not observe statistically significant changes of expression in any of the following genes: *PTK6*, *ARFRP1*, *EEF1A2*, *PPDPF*, *HELZ2*, *LIME1*, *TPD52L2*, *ZBTB46*, *ZGPAT*, *SLC2A4RG*, or *DNAJC5* (data are not shown). No detectable levels of the following genes were observed in the GBM cell lines used in our study: *KCNQ2* (potassium voltage‐gated channel subfamily Q member 2), *FNDC11*, or *ABHD16B*.

### 3.4 eQTL analysis

Table 1 summarizes the eQTL analysis results. *STMN3* was a significant eQTL with the candidate functional SNP rs3761124 in all three datasets. *RTEL1* was significant in both UCLA and TCGA data sets, but not in the CMC dataset. *GMEB2* was significant only in the UCLA dataset. Therefore, *STMN3* is a consistent eQTL for rs3761124 in early neurological development, in the normal adult brain and in glioma or during gliomagenesis, whereas the *RTEL1* expression correlated with rs3761124 only during early neurological development and in *IDH1* wild‐type glioma. Furthermore, *GMEB2* is essential during early neurological development.

**Table 1:** eQTL results of rs3761124: during early brain development (UCLA), in nondiseased adult brain (CMC), and *IDH1* wild‐type glioma (TCGA)

		CMC (*N* = 216)		UCLA (*N* = 219)		TCGA (*N* = 211)	
Gene ID	Gene Name	*β* (*SE*)†	*p*	*β* (*SE*)	*p*	*β* (*SE*)	*p*
ENSG00000197457.5	*STMN3*	**−.07 (0.02)**	**1.85 E−4**	**−.04 (0.01)**	**2.38 E−3**	**−.04 (0.02)**	**1.5 E−02**
ENSG00000125508.3	*SRMS*	.01 (0.05)	8.20 E−1	N/A^a^	N/A^a^	.02 (0.05)	7.50 E−1
ENSG00000258366.3	*RTEL1*	−.01 (0.03)	6.32 E−1	**.06 (0.02)**	**1.56 E−2**	**.06 (0.03)**	**3.07 E−2**
ENSG00000026036.16	*RTEL1‐TNFRSF6B*	.03 (0.03)	2.70 E−1	−.01 (0.03)	6.89 E−1	−.03 (0.03)	3.20 E−1
ENSG00000101216.6	*GMEB2*	−.01 (0.02)	3.77 E−1	**−.06 (0.01)**	**8.38 E−7**	.02 (0.01)	1.23 E−1

*Note*: Listed results were of the candidate target genes identified by RT‐PCR assays after CRISPR−cas9 deletion of Enhancer 2. Values in bold represent genes that showed significant association with the functional SNP rs3761124 in the analyzed datasets.

Abbreviations: CMC, CommonMind Consortium; RT‐PCR, real‐time polymerase chain reaction; TCGA, The Cancer Genome Atlas; UCLA, University of California, Los Angeles.

a This gene did not pass RNA‐seq Q/A in the UCLA dataset.

### 3.5 Colocalization of eQTL and GICC GWAS meta‐analysis summary statistics

Using the 1000 Genome European population as a reference panel, COLOC results showed that the PP3 is 0 and PP4 is 0.82 for colocalization of eQTL (CMC dataset: target gene *STMN3*) and the corresponding GICC GWAS meta‐analysis summary statistics. This suggests that rs3761124 has high probability of being a causal variant for both the eQTL and glioma GWAS signals.

### 3.6 Visualization of Hi‐C chromosome conformation interactions

**Figure 5:**
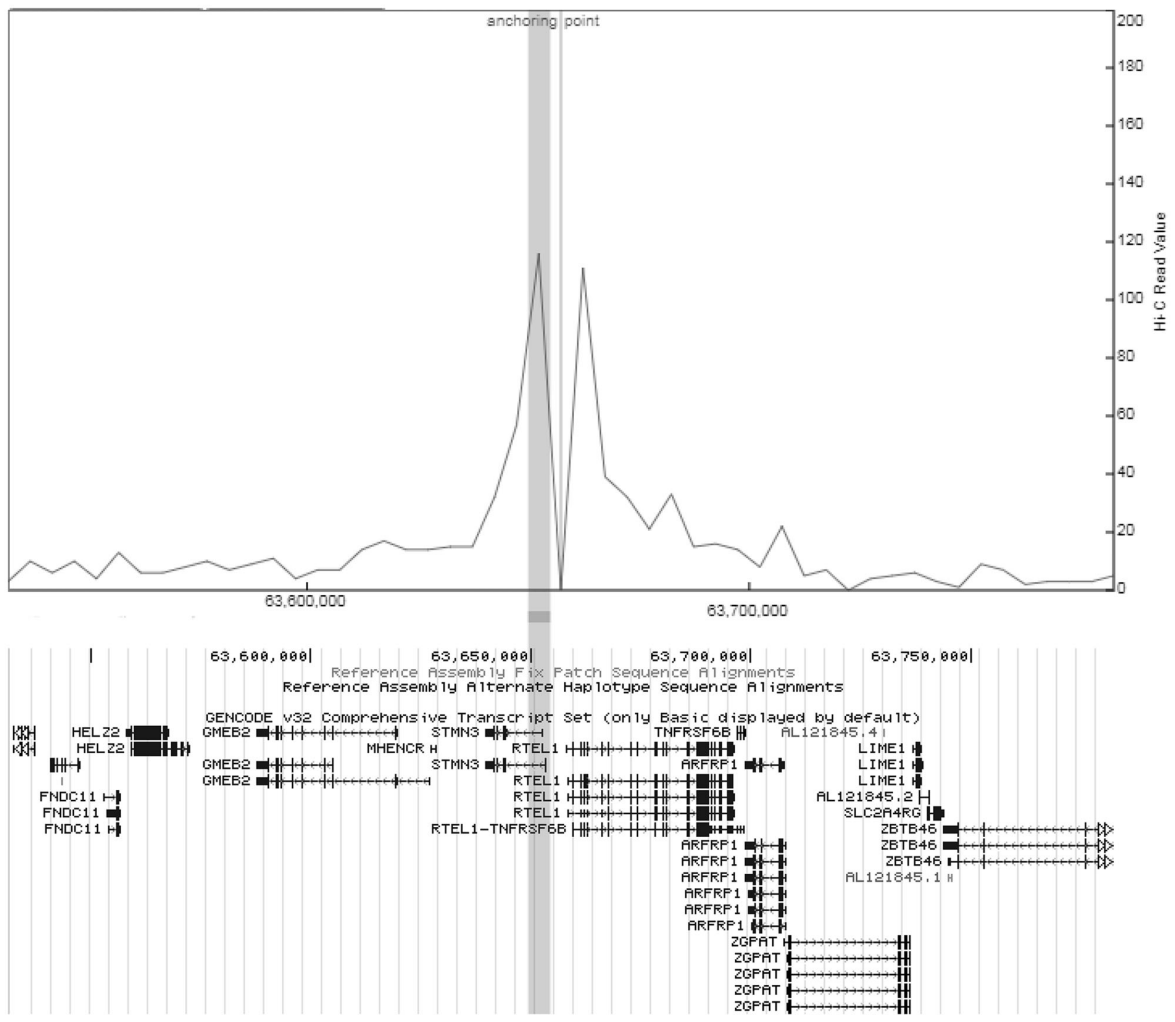
Visualization of chromosome interactions with rs3761124 in 20q13.33. Virtual 4C was constructed from Hi‐C data of the GBM cell line, G583. The anchoring point was the location of rs3761124 and also the bait region. Interactions of the bait with genomic regions were highlighted as peaks in the virtual 4C presentation. The horizontal bar shows the location of the promoter of *STMN3* and corresponds to its interaction peak (contained within the vertical bar). Another interaction peak is observed near the promoter of *RTEL1*, which is transcribed in the opposite direction of *STMN3*,to the right of the anchoring point. GBM, glioblastoma multiforme

The virtual 4C constructed from Hi‐C data generated from the GBM cell line, G583, is illustrated in Figure 5. It shows interactions, demonstrated as peaks, between the region containing rs3761124 and promoters of *STMN3* and *RTEL1*.

## 4 DISCUSSION

We provide evidence that rs3761124 is a functional SNP on 20q13.33 mapping to a risk enhancer using cell‐based enhancer activity assays. We show that rs3761124 had allele‐specific effects on enhancer activity in the forward direction only. We previously noted unidirectional, rather than bidirectional, allele‐specific effects on enhancer activity in colorectal cancer functional studies (Biancolella et al., 2014; Fortiniini et al., 2014). We further show that this SNP affects glioma risk potentially through the altered expression of *STMN3, RTEL1*, *GMEB2*, and several other genes based on CRISPR deletion of the risk enhancer. The complementary methods of eQTL mapping and Hi‐C interaction data support *STMN3*, being the most consistent target gene across biological models, whereas *RTEL1* and *GMEB2* expression correlated with rs3761124 only in glioma and/or during early neurological development but not in the adult normal brain.

The identification of multiple target genes suggests that this putative risk enhancer interacts with multiple promoters, some of which may also depend on additional molecular stimuli. It is not unprecedented that there may be multiple gene targets of a risk enhancer. A previous report identified rs73001406 as a candidate functional variant for glioma on 11q23.3, with *PHLDB1* and *DDX6* as potential target genes (Baskin et al., 2015). We previously described a risk enhancer for colorectal cancer on 11q23.1 that correlated with the expression of three target genes (Biancolella et al., 2014). Other studies reported multiple gene targets of risk enhancers in cancers, such as prostate cancer (Huang et al., 2014) and breast cancer (Betts et al., 2017; Dunning et al., 2016; Ghoussaini et al., 2016).

*STMN3* (also known as *SCLIP* or SCG10‐like protein) is one of the members of the stathmin family of proteins that plays an important role in the regulation of microtubule stability (Charbaut et al., 2001). In addition to our own finding, the importance of *STMN3* in glioma is supported by other studies. For example, a recent transcriptome‐wide association study (TWAS), which used the Genotype‐Tissue Expression Project (GTEx) data to build a gene expression model, identified *STMN3* as a highly significant gene associated with the risk of adult glioma (4.54 × 10^‐27^; Atkins et al., 2019). Among 55 adult tissues analyzed through GTEx, the *STMN3* expression is the highest in the 13 CNS tissues (GTEX portal access 18 June, 2020); furthermore, messenger RNA (mRNA) and protein of this gene are overexpressed in human glioma of all grades as compared with normal brain tissues (Zhang et al., 2015). The overexpression of *STMN3* increased growth and mobility of glioblastoma cells, whereas *STMN3* knockdown impaired cell growth, proliferation, invasion, and migration (Zhang et al., 2015). Another study reported that high‐resolution chromosome conformation capture (Hi‐C) data generated in H1 embryonic stem cell and neuronal progenitor cell lines revealed a physical interaction between the *STMN3* promoter and the top GWAS SNP rs2297440 (Dixon et al., 2015; Labreche et al., 2018), which is in high LD (*r* ^2^ = .92) with the functional SNP rs3761124. Therefore, among the several target genes identified in this study, *STMN3* appears to be the most robust and consistently validated.

Our data also implicate *RTEL1* and *GMEB2* in glioma, but their role may be more context‐specific. CRISPR deletion of the risk enhancer containing rs3761124 in the U‐87 MG and LN‐229 GBM cell lines (which are both *IDH1* wild type) correlated with altered expression of both genes. Consistent with this, rs3761124 correlated with altered expression of *RTEL1* in *IDH1* wild‐type glioma and during early brain development but not in the CMC brain tissues. This lack of supportive evidence for this gene in the adult normal brain is also seen in a recent TWAS study, which showed *RTEL1* was not a significant target gene using the GTEx adult nondisease brain tissues (Atkins et al., 2019). As *RTEL1* is a DNA helicase that maintains genomic stability directly by suppressing homologous recombination, it may be quiescent during normal adult brain, but becomes increasingly active during conditions of active cellular growth, such as gliomagenesis and/or during early neurological development. Therefore, our candidate functional variant rs3761124 may impact genomic stability through changes in activity of its risk enhancer, modulating the expression of *RTEL1*.

Another target gene found in selective context is *GMEB2*, which is a transacting factor that binds to glucocorticoid modulatory elements (GME) present in the *Tyrosine Aminotransferase* promoter, increasing sensitivity to glucocorticoid (Oshima et al., 1995). *GMEB2* has been associated with prostate cancer, but its role in gliomagenesis is unknown. Nevertheless, dexamethasone, a common corticosteroid with a high glucocorticoid activity, has been found to significantly increase invasion, cell proliferation, and angiogenesis in vitro or in vivo in GBM stem cell lines, including GBM stem cells that are *IDH1* wild‐type (Luedi et al., 2018). Therefore, it is possible that *GMEB2* may be a target gene in the brain only during conditions of cellular proliferation, such as at the time of early neurological development and during gliomagenesis.

Two additional genes were identified as potential target genes of the putative risk enhancer on 20q13.33 after CRISPR−Cas9 deletion including *RTEL1‐TNFRSF6B* and *SRMS*. Neither of these genes was identified in any of our eQTL analyses. *RTEL1‐TNFRSF6B* is a noncoding, readthrough transcript that is subject to NMD (Chang et al., 2007), but it is currently unclear how a change in mRNA decay will affect glioma development. *SRMS* belongs to a family of nonreceptor tyrosine kinases that have been involved in a number of cancers, including fibrosarcoma (Lin et al., 2013), eosinophilic variant of chromophobe renal cell carcinoma (Pagano et al., 2018), and breast cancer (Fan et al., 2015), but its function in gliomagenesis remains unknown. Additional studies will be required to determine if these genes are indeed relevant to glioma risk.

There are some limitations to our study. We cannot discount the possibility that there are additional functional SNPs on 20q13.33. Although we used all available publicly available datasets to identify candidate enhancers, these may not have captured all relevant enhancers across this region. In addition, we restricted our analysis to SNPs with an *r* ^2^ > .6 (in CEU population) to the index SNP, and we may have missed functional SNP(s) that would be captured at a lower *r* ^2^. Our in vitro assessment of enhancer activity was conducted in only two GBM cell lines and we do not know if the candidate enhancers that showed no activity in these cells would have been active in other cell lines. Similarly, our CRISPR deletion experiments were conducted in GBM cell lines and additional/different target genes of this enhancer may be seen in more relevant “normal” cells of the brain. Finally, our eQTL analyses were restricted to data available, which may not capture all relevant cellular contexts to capture associations with the functional SNP. Despite this, we believe we provide strong supportive evidence for the identification of at least one functional SNP relevant to gliomagenesis on 20q13.33.

In summary, we report identification and characterization of a functional SNP, rs3761124, that affects the activity of an enhancer on 20q13.33 that leads to modulated expression of multiple genes implicated in glioma risk. Our results are concordant with reports by others that a multiple‐gene, rather than a single‐gene, association with GBM is present at 20q13.33 (Atkins et al., 2019). As the effect of these genes on glioma growth and development has not been evaluated in depth, further studies to evaluate their molecular mechanisms may lead to novel therapeutic strategies in the future.

## CONFLICT OF INTERESTS

The authors declare that there are no conflict of interests.

## AUTHOR CONTRIBUTIONS

Rose K. Lai and Graham Casey contributed to conception and design. Mourad Wagdy Ali, Sarah J. Plummer, and Christopher H. Dampier performed and analyzed luciferase assays. Mourad Wagdy Ali, Cem Kuscu, and Mazhar Adli conceived and performed CRISPR‐Cas9 experiments. C. Pawan K. Patro performed eQTL mapping. Mourad Wagdy Ali, Graham Casey, C. Pawan K. Patro, and Rose K. Lai wrote the manuscript with input from all authors. All authors read and approved the manuscript and gave consent to publication.

## WEB RESOURCES

The enhancer region analysis was performed using University of California Santa Cruz Genome Browser: http://genome.ucsc.edu/. ChIP‐Seq peaks for histone modifications were obtained from the Encyclopedia of DNA Elements: https://www.encodeproject.org/. CRISPR gRNAs (guide RNAs) were designed using https://www.crisprscan.org/. eQTL mapping was done using CommonMind Consortium (CMC) release 1 (http://commonmind.org/), University of California Los Angeles postconception fetal tissues collection (https://labs.dgsom.ucla.edu/geschwind/pages/eqtl-browser), and The Cancer Genome Atlas GBM and lower grade glioma (LGG) cohorts (https://www.cancer.gov/about-nci/organization/ccg/research/structural-genomics/tcga). The GICC GWAS summary statistics is available at European Genome‐phenome Archive (http://www.ebi.ac.uk/ega/; accession number: EGAS00001003372).

## Data Availability

The data that support the findings of this study are available from the corresponding author upon reasonable request.
